# The Eicosapentaenoic Acid Metabolite 15-Deoxy-δ^12,14^-Prostaglandin J_3_ Increases Adiponectin Secretion by Adipocytes Partly *via* a PPARγ-Dependent Mechanism

**DOI:** 10.1371/journal.pone.0063997

**Published:** 2013-05-29

**Authors:** Jennifer Lefils-Lacourtablaise, Mairobys Socorro, Alain Géloën, Patricia Daira, Cyrille Debard, Emmanuelle Loizon, Michel Guichardant, Zury Dominguez, Hubert Vidal, Michel Lagarde, Nathalie Bernoud-Hubac

**Affiliations:** 1 Université de Lyon, Institut National de la Santé et de la Recherche Médicale Unité Mixte de Recherche 1060, “Laboratoire de Recherche en Cardiovasculaire, Métabolisme, Diabétologie et Nutrition”, Institut Multidisciplinaire de Biochimie des Lipides/Institut National des Sciences Appliquées-Lyon, Villeurbanne, France; 2 Laboratorio de Biología Celular del Endotelio, Instituto de Medicina Experimental-Facultad de Medicina Universidad Central de Venezuela, Caracas, Venezuela; Tohoku University, Japan

## Abstract

The intake of ω-3 polyunsaturated fatty acids (PUFAs), which are abundant in marine fish meat and oil, has been shown to exert many beneficial effects. The mechanisms behind those effects are numerous, including interference with the arachidonic acid cascade that produces pro-inflammatory eicosanoids, formation of novel bioactive lipid mediators, and change in the pattern of secreted adipocytokines. In our study, we show that eicosapentaenoic acid (EPA) increases secreted adiponectin from 3T3-L1 adipocytes and in plasma of mice as early as 4 days after initiation of an EPA-rich diet. Using 3T3-L1 adipocytes, we report for the first time that 15-deoxy-δ^12,14^-PGJ_3_ (15d-PGJ_3_), a product of EPA, also increases the secretion of adiponectin. We demonstrate that the increased adiponectin secretion induced by 15d-PGJ_3_ is partially peroxisome proliferator-activated receptor-gamma (PPAR-γ)-mediated. Finally, we show that 3T3-L1 adipocytes can synthesize 15d-PGJ_3_ from EPA. 15d-PGJ_3_ was also detected in adipose tissue from EPA-fed mice. Thus, these studies provide a novel mechanism(s) for the therapeutic benefits of ω-3 polyunsaturated fatty acids dietary supplementation.

## Introduction

Eicosapentaenoic (EPA) and docosahexaenoic (DHA) acids are ω-3 polyunsaturated fatty acids (PUFA), found primarily in marine lipids, that display many health benefits, such as the improvement of insulin sensitivity with beneficial effects against obesity and the prevention of cardiovascular diseases [1–4]. The American Heart Association recommends eating fish rich in ω-3 fatty acids. Despite numerous studies suggesting protective actions of EPA and DHA, the cellular and molecular rational for their intake remains of considerable interest.

It is assumed that these beneficial effects are linked to the ability of both acids to inhibit the production of ω-6 PUFA-derived prostaglandins and leukotrienes [5]. Additionally, recent studies have shown that a series of novel ω-3 PUFA-derived compounds could be responsible for eliciting their beneficial effects [6–8]. Resolvins and protectins have been shown for example to display potent anti-inflammatory and immunoregulatory actions [9, 10].

Among bioactive lipid mediators, prostaglandins (PG) exert a plethora of biological activities. PGs of the 2-series are formed by cyclooxygenase (COX)-1 and COX-2 from arachidonic acid (AA). COX converts AA (released from membrane phospholipids through the activity of several phospholipases, mainly phospholipases A_2_) to the unstable cyclic endoperoxide intermediates PGG_2_/H_2_ [11]. PGH_2_ is subsequently metabolized to several prostanoids, PGD_2_, PGE_2_, PGF_2α_, PGI_2_ and thromboxane A_2_ (TXA_2_) through the action of synthases (prostaglandin D synthase [PGDS], PGES, PGFS, PGIS and TXAS) [12,13]. *In vitro*, PGD_2_ spontaneously dehydrates to PGJ_2_ [14] which is converted to 15-deoxy-δ^12,14^-PGJ_2_ (15d-PGJ_2_) in the absence of albumin [15]. 15d-PGJ_2_ has been detected *in vivo* [15,16] and has been shown to exhibit *in vitr*o and *in vivo* anti-inflammatory and anti-proliferative effects [15,17]. The anti-inflammatory cyclopentenone PGs exert their effects, in part, by binding and activating the peroxisome proliferator-activated receptor-gamma (PPAR-γ) [18,19].

EPA can also be enzymatically converted by cyclooxygenase into PGH_3_ which in turn is converted to the 3-series PGs, e.g., PGD_3_, PGE_3_, PGF_3α_ and PGI_3_ [20–22]. The eicosanoids derived from EPA have less inflammatory activities compared with those produced from AA [23–25].

Another mechanism by which ω-3 PUFA may exert beneficial effects is by modulating the secretion of adipocytokines [26, 27]. Adiponectin is one of the most abundant plasma protein adipocytokines that shows anti-inflammatory, anti-atherogenic and insulin-sensitizing properties [28, 29]. The potential mechanism by which ω-3 PUFA modulate adiponectin secretion is not fully understood, but may partially involve PPAR-γ [30-33] which has been shown to play an important role in the transcriptional activation of the adiponectin gene [34].

**Figure 1:**
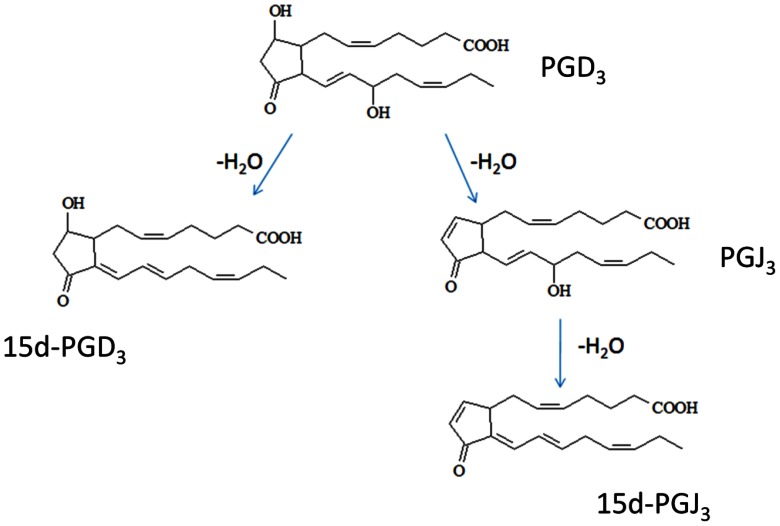
Proposed pathway for PGD_3_ metabolism (adapted from Ref Shibata et al., 2002 for PGD_2_).

A recent study showed the formation of J-series PGs from EPA [35]. The pathway by which 15d-PGJ_3_ could be generated is shown in Fig. 1. PGD_3_ would be first dehydrated to 15d-PGD_3_ and PGJ_3_ and then the latter compound would be directly converted to 15d-PGJ_3_.

We concomitantly raised the possibility that 3-series PGs, PGD_3_ and J_3_ PGs might influence the production of adipokines. Our studies show that EPA, PGD_3_ and 15d-PGJ_3_ increased adiponectin secretion by 3T3-L1 and that this partly occurred *via* a PPAR-γ-dependent mechanism. Moreover, we present evidence that 15d-PGJ_3_ is formed in significant amount after incubation of cells with EPA.

## Materials and Methods

Ethics Statement. This study was carried out in strict accordance with the European Communities Council Guidelines (November 24, 1986, 86/609/EEC) and all animal experiments followed a strict protocol. This study was specifically approved by the Committee on the Ethics of Animal Experiments of the INSA of Lyon CETIL (permit Number: 012012). All efforts were made to minimize suffering.

### Materials

3T3-L1 cells were obtained from the American Type Culture Collection (ATCC, Manassas, VA, USA). Dexamethasone, 3-isobutyl-1-methyl-xanthine and GW9662 were purchased from Sigma-Aldrich (Saint Quentin Fallavier, France). Insulin was obtained from Novo Nordisk Actrapid and rosiglitazone from Molekula (La Tour du Pin, France). EPA, as the synthetic triglyceride, Omegavie 90, was purchased from Polaris (Pleuven, France). Mouse adiponectin EIA was purchased from SpiBio (Montigny Le Bretonneux, France). EPA, d_5_-EPA, PGD_3_ and PGD_2_ were purchased from Cayman Europe (Tallinn, Estonia). All solvents used were of HPLC quality. RNeasy mini kit and rotor-Gene Q were from Qiagen (Courteboeuf, France). Superscript II was from Invitrogen (Eragny, France). Random hexamers and oligo (dT) primers were from Promega (Charbonnières, France). XBridge™ columns were from Waters (St Quentin, France).

### Cell Culture

3T3-L1 preadipocytes were cultured in a 5% CO_2_ atmosphere at 37°C in a growth medium containing the following constituents: Dulbecco’s modified Eagle’s medium (DMEM) supplemented with 10% fetal calf serum, 4 mM L-glutamin and antibiotics. Differentiation of the cells was induced after confluence using the growth medium containing 0.5 mM 3-isobutyl-1-methyl-xanthine, 5 µg/mL insulin, 10 µmol/L rosiglitazone and 0.25 µmol/L dexamethasone. On day 2, the media was replaced by the growth medium containing 5 µg/mL insulin and 10 µmol/L rosiglitazone for 2 days. The fully differentiated phenotype was controlled by observing the cells using light microscopy for the existence of the typical appearance of extensive accumulation of lipid droplets. Insulin was removed on day 4 by changing the media to growth medium containing 10 µmol/L rosiglitazone and cells were maintained thereafter in this medium. Day 10 differentiated 3T3-L1 adipocytes were used for the experiments.

### Effects of Eicosapentaenoic Acid and Prostaglandins of the 3 Series on Adiponectin Secretion

Preceding the different treatments, 3T3-L1 cells were washed with phosphate-buffered saline (PBS) and incubated under serum-free culture medium for 4 h. Cells were then incubated in fresh DMEM for 2 and 4 h with EPA (1 µM or 10 µM) complexed with bovine serum albumin (50 µM) or with PGD_3_ (1 µM) or 15d-PGJ_3_ (100 nM) in an ethanolic solution in the presence or absence of 10 µM GW9662, a PPAR-γ antagonist. Control cells received vehicle (bovine serum albumin or ethanol alone).

### Secreted Adiponectin Protein Measurement

Concentrations of adiponectin in the cell culture media and in sera were measured by enzyme-linked immunosorbent assay (ELISA) following the manufacturer’s instructions. In brief, samples were incubated in a 96-well pre-coated plate with a monoclonal antibody specific of mouse adiponectin for 1 h at room temperature. After washing the plate, anti-adiponectin antibody horseradish peroxidase tracer was added. Incubation was carried out for 1 h and substrate solution was then added for 10 min after washing the plate. The reaction was arrested by addition of stop solution. The concentration of the adiponectin was determined by spectrophotometry.

### Quantitative PCR

RNAs were prepared using the RNeasy mini kit according to the method recommended by the manufacturer. First-strand cDNAs were synthesized from 1 µg of total RNA in the presence of 100 units of Superscript II using a mixture of random hexamers and oligo (dT) primers. Real-time PCR assays were performed using a Rotor-Gene Q as described previously [36]. The following primers were used : for FAS: forward 5′-GTGCACCCCATTGAAGGTTCC-3′, reverse 5′- GGTTTGGAATGCTGTCCAGGG-3′; for adiponectin: forward 5′- AGGCCGTGATGGCAGAGATG-3′, reverse 5′- CTTCTCCAGGTTCTCCTTTCCTGC-3′; for FABP4 forward 5′-CAGAAGTGGGATGGAAAGTCG-3′, reverse 5′- CGACTGACTATTGTAGTGTTTGA-3′; for PPAR-γ: forward 5′-TCTCTCCGTAATGGAAGACC-3′, reverse 5′-GCATTATGAGACATCCCCAC-3′; for PDK4: forward 5′-AGTGTGCAAAGATGCTCTGC-3′, reverse 5′- AGAGCATGTGGTGAAGGTGTG-3′. Glucuronidase, beta (Gusb) mRNA level was used to normalize the data.

### Animals and Diets

Ethics Statement. Mice were treated in accordance with the European Communities Council Guidelines (November 24, 1986, 86/609/EEC) and all animal experiments followed a strict protocol, approved by the Committee on the Ethics of Animal Experiments of the INSA of Lyon CETIL (permit Number: 012012). Animals were kept on a 12∶12-h light/dark cycle and were allowed an unrestricted access to diet and water. Animals were randomised into groups of 8 and assigned to a standard control diet A03 (SAFE, Augy, France) containing 5% lipids, or to an EPA-enriched diet reconstituted with lipid-free powder (SAFE, Augy, France), 4.5% sunflower oil (Lesieur, Asnières-sur-Seine, France) and 0.5% 1,2,3-trieicosapentaenoylglycerol (Polaris, Pleuven, France) for 4 days. The complete nutrient and fatty acid composition of each of the diets are listed in Table 1. The diet was replaced every day for a new one to avoid EPA peroxidation. Mice were killed by lethal intraperitoneal injection of pentobarbital on day 0 and day 4 and blood was collected and immediately centrifuged. Plasma aliquots were sampled and stored at −80°C before measurement of adiponectin. Adipose tissue was dissected and immediately frozen in liquid nitrogen and then stored at −80°C until analysis of 15d-PGJ_3_.

**Table 1:** Nutrient composition and fatty acid composition of standard (A03) and EPA-rich diet.

	standard (A03)	EPA-rich
**Nutrients**		
Carbohydrates	51.7	52.2
Proteins	21.4	19.0
Lipids	5.0	5.0
Minerals	5.7	6.6
Fibers	3.9	5.0
Humidity	12.2	12.0
**Fatty acids**		
16∶0	22	5
16∶1	2	tr
18∶0	11	3
18∶1	26	39
18∶2	38	41
18∶3	tr	tr
20∶5	nd	10

tr: trace. nd: non detected.

### Dehydration/isomerization of PGD_2_ and PGD_3_

PGD_2_ or PGD_3_ (1 mM) were incubated in PBS at 37°C. After acidification to pH 3, samples were extracted three times with 4 volumes of ethyl acetate and extracts were dried under nitrogen gas. The samples were reconstituted in ethanol.

### HPLC Separation of PGD_2_ and PGD_3_ Metabolites

Products from PGD_2_ or PGD_3_ were isolated by HPLC using a 4.6×250 mm C18 XBridge™ column. The solvent system used was a gradient consisting of acetonitrile/water acidified to pH 3 (2/8, v/v)) (solvent A) to acetonitrile (solvent B). The flow rate was 1 mL/min beginning at 100% A, followed by an increase to 100% B over 30 min. The column was then washed with 100% B for 10 more min. The elution profiles were monitored by UV absorbance at 195 nm.

### Analysis of 15d-PGJ_3_ by Gas Chromatography (GC)-mass Spectrometry (GC-MS) and GC/tandem MS

15d-PGJ_3_ putative compound eluting as a single peak from HPLC was collected. The compound was converted to a pentafluorobenzyl ester derivative, with pentafluorobenzyl bromide and diisopropylethylamide in acetonitrile. A Thermo Trace GC connected to a Thermo PolarisQ MS operated in negative ion chemical ionization (NICI) mode was used to analyze the sample. 15d-PGJ_3_ was detected by GC-MS using selected ion monitoring for the [M-CH_2_C_6_F_5_]^-^ ion (*m/z* 313). The molecular ion *m/z* 313 of putative 15d-PGJ_3_ was subjected to collision-induced dissociation (CID).

### Analysis of 15d-PGJ_3_ and d_5_-15d-PGJ_3_ in the Culture Medium of 3T3-L1 Cells Incubated with EPA or d_5_-EPA

Cells were incubated with EPA or d_5_-EPA. Products were separated by HPLC; the HPLC fraction supposed to contain 15d-PGJ_3_ or d_5_-15d-PGJ_3_ was collected and was analyzed by GC-MS and GC-MS/MS as described above. The molecular ions *m/z* 313 of putative 15d-PGJ_3_ and *m/z* 318 of putative d_5_-15d-PGJ_3_ were subjected to collision-induced dissociation (CID). Spectra that are shown were obtained at 2 eV. Quantifications were performed using d_4_-15d-PGJ_2._

### Analysis of 15d-PGJ_3_ in Adipose Tissue from EPA Fed-mice

Epididymal adipose tissues were obtained from previously mentioned feeding study. Sample was extracted using a silica Sep-Pak cartridge. 15d-PGJ3 was purified by HPLC as described above, converted into a pentafluorobenzyl ester derivative, and analyzed by GC-MS/MS as described above.

### Statistical Analysis

Statistical analyses were performed using Student’s *t*-test. The difference was considered significant at *p*<0.05. The results are expressed as means ± sem.

## Results

### Increased Plasma and Media Adiponectin Concentrations in Mice Fed an EPA-enriched Diet and in 3T3-L1 Cells Treated with EPA, Respectively

**Figure 2:**
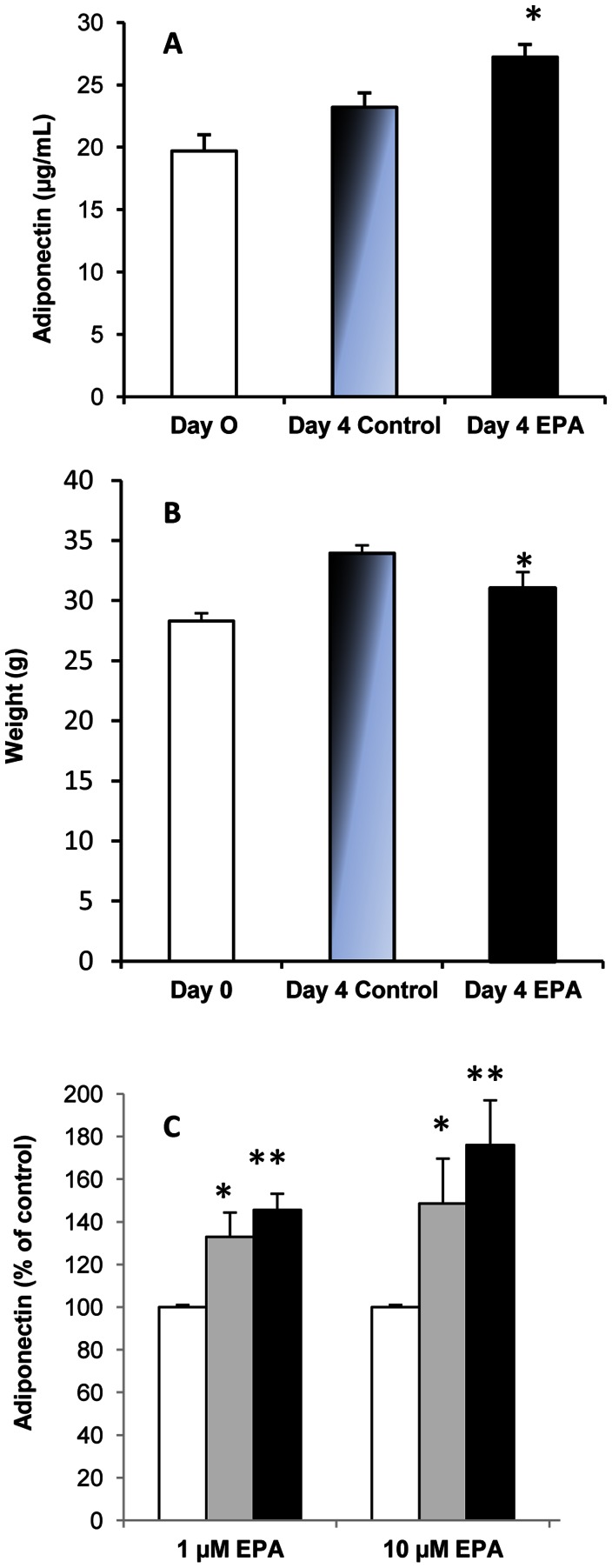
(A) Plasma levels of adiponectin from mice fed a standard diet or an EPA-rich diet. Plasma levels are expressed in µg/mL. Results are means ± sem (n = 8). *P<0.05 as compared to the control group. **(B) Body weight gain (g) of mice fed a standard diet or an EPA-rich diet.** Mice were killed on days 0 and 4. Results are means ± sem (n = 8). *P<0.05 as compared to the control group. **(C) Effects of eicosapentaenoic acid on adiponectin secretion by 3T3-L1 adipocytes.** Cells were incubated for 2 h (gray) or 4 h (black) with 1 µM or 10 µM EPA complexed with bovine serum albumin. Adiponectin in the medium was determined by ELISA. Results are means ± sem (n = 4 in triplicate), expressed as percentage of the control (□). Statistical significance is represented as *P<0.05 *vs* control.

We examined the effects of EPA on the production of adiponectin in mice fed an EPA-enriched diet. Plasma level of adiponectin was significantly increased (+17%) as early as 4 days after initiation of the EPA-enriched diet group compared to control mice (Fig. 2 A).

On the contrary, blood leptin secretion was significantly decreased (by 1.5-fold) after 4 days of the EPA-enriched diet feeding (not shown). We also observed that the body weight gain of mice fed the EPA-enriched diet was significantly lower than that of mice fed the standard diet (31.0 g +/−1.4 *vs* 33.9+/−0.7) (Fig. 2 B). We also examined the effects of EPA on the adiponectin concentration in the culture media of cells. 3T3-L1 cells were incubated for 2 h or 4 h with 1 µM or 10 µM of EPA. As shown in Fig. 2 C, EPA induced a significant increase in adiponectin secretion.

### Synthesis of 15d-PGJ_3_ from PGD_3_ in Phosphate-buffered Saline

**Figure 3:**
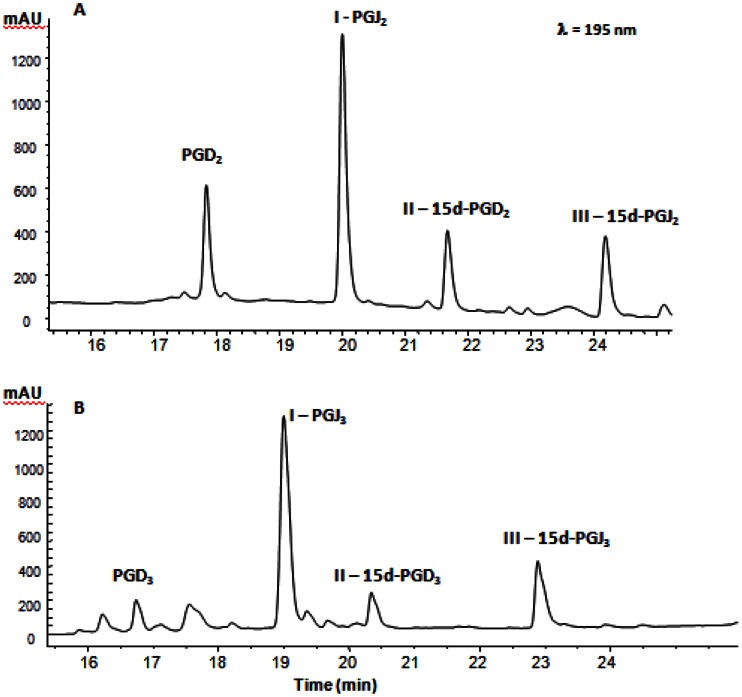
HPLC profile of metabolites formed from PGD_2_ (A) or PGD_3_ (B). 1 mM of PGD_2_ or PGD_3_ was incubated in phosphate-buffered saline at 37°C for 24 h. PGD_2_/PGD_3_ and their metabolites were chromatographed on a Waters Xbridge C18 column (4.6×250 mm, 3.5 µm) at a flow rate of 1 ml/min starting at 100% solvent A (acetonitrile/water acidified to pH 3, 2/8 v/v) to 100% solvent B (acetonitrile) from 1 to 30 min. The elution profiles were monitored by UV absorbance at 195 nm.

**Figure 4:**
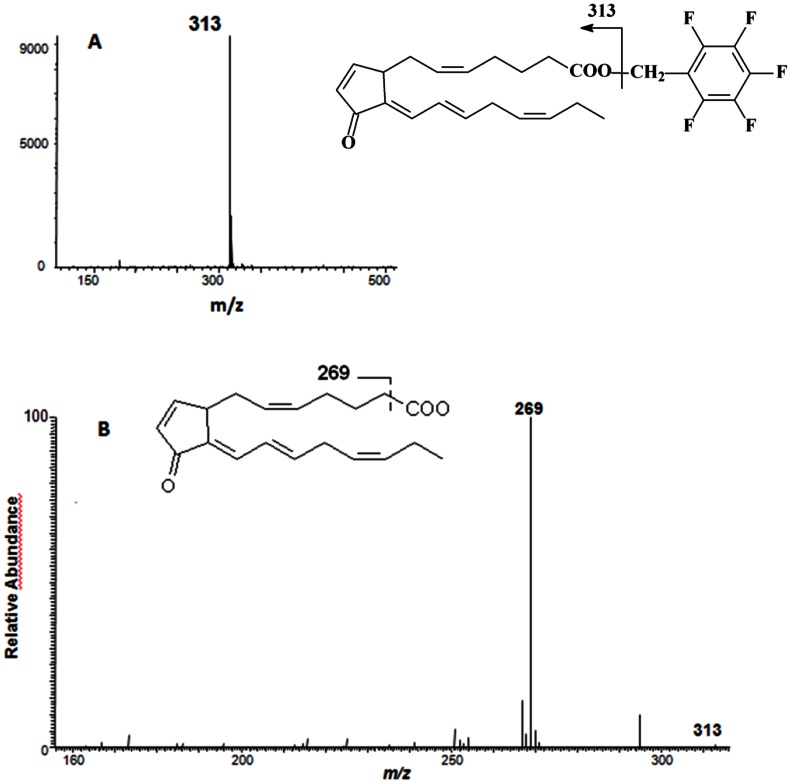
GC-MS (A) and GC-MS/MS (B) spectra of 15d-PGJ_3_ generated during incubation of PGD_3_ in phosphate-buffered saline. The anion at *m/z* 313 [M-CH_2_C_6_F_5_]^-^ generated by NICI was subjected to CID. The structure of the derivative and the proposed structure of the product ion produced by CID of *m/z* 313 are shown in the figure.

Previously, it has been shown that PGD_2_, a prostaglandin derived from AA, is converted sequentially to J_2_ prostaglandins *in vitro* [14, 15]. Shibata et al [15] showed that PGD_2_ is initially converted to the dehydration products 15d-PGD_2_ and PGJ_2,_ the latter being converted to 15d-PGJ_2_. A recent study showed that J_3_ prostanoids are also formed *in vitro* from PGD_3_ [35]. PGD_2_ or PGD_3_ was incubated in PBS at 37°C for 72h and the products were analyzed by HPLC. By comparison with PGs of the 2-series (formed from PGD_2,_ Figure 3A) or commercial (not shown), peaks I, II and III were assumed to be PGJ_3_, 15d-PGD_3_ and 15d-PGJ_3_ (Fig. 3B, peaks I, II and III, respectively). To further substantiate the structural identity of peak III as 15d-PGJ_3_, the HPLC fraction containing this peak was collected and analyzed by GC-MS and GC-MS/MS. The predicted unique [M-CH_2_C_6_F_5_]^-^ ion for the pentafluorobenzyl ester derivative 15d-PGJ_3_ is *m/z* 313. The GC/NICI/MS analysis of the product chromatogram only showed a *m/z* 313 peak compatible with the formation of 15d-PGJ_3_ (Fig. 4A). This peak was analyzed by CID and the CID spectrum is shown in figure 4B. A predicted fragment, *m/z* 269 [M-CH_2_C_6_F_5_-CO_2_]^-^, was obtained.

### Exposure of 3T3-L1 Adipocytes to PGD_3_ and 15d-PGJ_3_ Leads to Increased Adiponectin Levels

**Figure 5:**
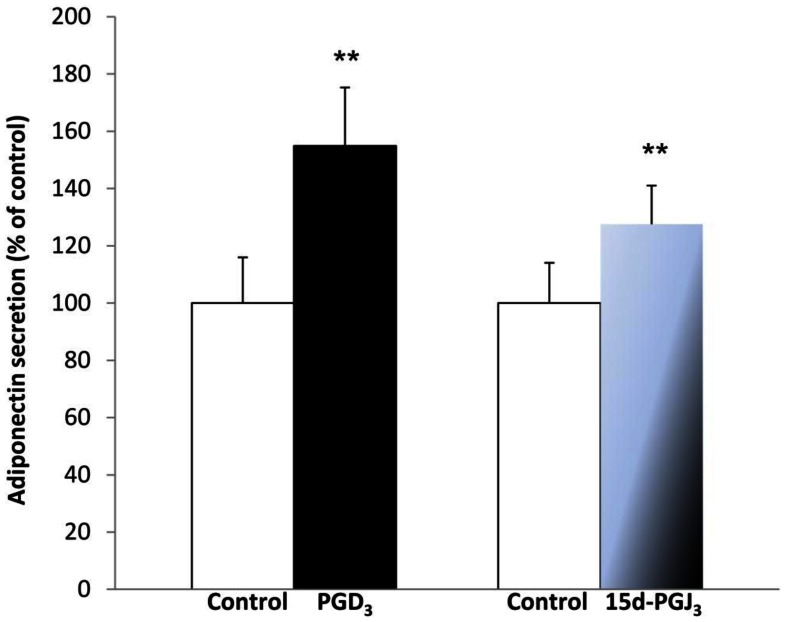
Effects of PGD_3_ and 15d-PGJ_3_ on adiponectin secretion by 3T3-L1 adipocytes. Cells were incubated for 2 h with 1 µM PGD_3_ or 0.1 µM 15d-PGJ_3_. Adiponectin in the medium was determined by ELISA. Results are means ± sem (n = 4 in triplicate), expressed as percentage of the control. Statistical significance is represented as **P<0.01 *vs* control.

We then examined whether PGD_3_ and 15d-PGJ_3_ could increase adiponectin secretion by 3T3-L1 adipocytes. Exposure of cells to 1 µM PGD_3_ and 100 nM 15d-PGJ_3_ resulted in increased adiponectin levels in 3T3-L1 adipocytes medium by 55% and 28%, respectively compared to control cells (Fig. 5). These concentrations were chosen since the increase in adiponectin secretion was observed from 1 µM EPA. We also observed an increased adiponectin secretion (+25%) after incubation of cells for 24 h with 100 nM 15d-PGJ_3_ compared to control cells (not shown).

### Formation of 15d-PGJ_3_ in Cell Medium after Incubation of Cells with EPA

**Figure 6:**
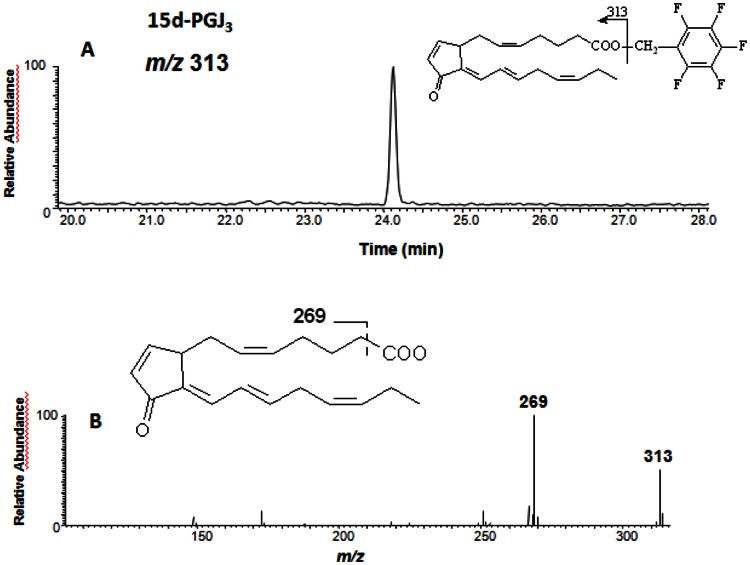
GC-MS and GC-MS/MS analysis of 15d-PGJ_3_ in the culture medium following incubation of cells with EPA. 3T3-L1 were incubated with 10 µM EPA for 4 h. Culture medium was extracted. 15d-PGJ_3_ was purified by HPLC. The 15d-PGJ_3_ HPLC peak was collected and was analyzed by GC-MS as a pentafluorobenzyl ester derivative (*m/z* 313) (Fig. 6A). B. GC-MS/MS spectrum of 15d-PGJ_3._ The molecular ion [M-CH_2_C_6_F_5_]^-^ of *m/z* 313 was subjected to CID. The structure of the derivative and the proposed structure of the product ion produced by CID of *m/z* 313 are shown in the figure.

**Figure 7:**
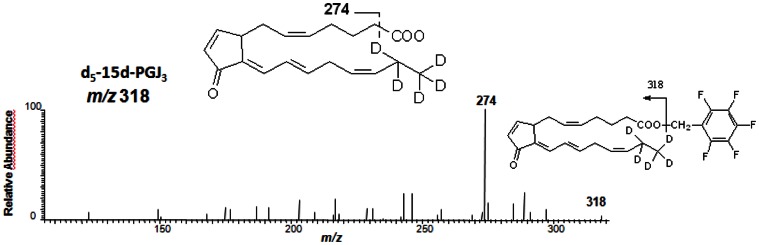
GC-MS/MS analysis of d_5_-15d-PGJ_3_ in the culture medium following incubation of cells with d_5_-EPA. 3T3-L1 cells were incubated with d_5_-EPA for 4 h. Culture medium was extracted. d_5_-15d-PGJ_3_ was purified by HPLC. The d_5_-15d-PGJ_3_ HPLC peak was collected and was analyzed by GC-MS/MS_._ The anion at *m/z* 315 [M-CH_2_C_6_F_5_]^-^ was subjected to CID. The structure of the derivative and the proposed structure of the product ion produced by CID of *m/z* 315 are shown in the figure.

We then sought to determine whether 15d-PGJ_3_ could be detected in the culture medium of cells incubated with EPA. 3T3-L1 were incubated with 10 µM EPA. Products were separated by HPLC; the HPLC fraction supposed to contain 15d-PGJ_3_ was collected and 15d-PGJ_3_ was analyzed by GC-MS and GC-MS/MS. As shown in Fig. 6A, a significant amount of 15d-PGJ_3_ was detected in the culture medium (nanomolar concentration). The observed production, *m/z* 269, generated by CID of the parent ion [M-CH_2_C_6_F_5_]^-^, is consistent with fragmentation of 15d-PGJ_3_ ([M-CH_2_C_6_F_5_-CO_2_]^-^) (Fig. 6B). To further substantiate the formation 15d-PGJ_3_ from EPA in 3T3-L1, cells were incubated with d_5_-EPA. As described above, products were separated by HPLC and the HPLC fraction containing the putative d_5_-15d-PGJ_3_ was collected and then analyzed by GC-MS/MS. The predicted molecular ion for [M-CH_2_C_6_F_5_]^-^ of d_5_-15d-PGJ_3_ is *m/z* 318. CID of m/z 318 generates the product ion at *m/z* 274 ([M-CH_2_C_6_F_5_-CO_2_]^-^) (Fig 7).

### Formation of 15d-PGJ_3_ *in vivo*

**Figure 8:**
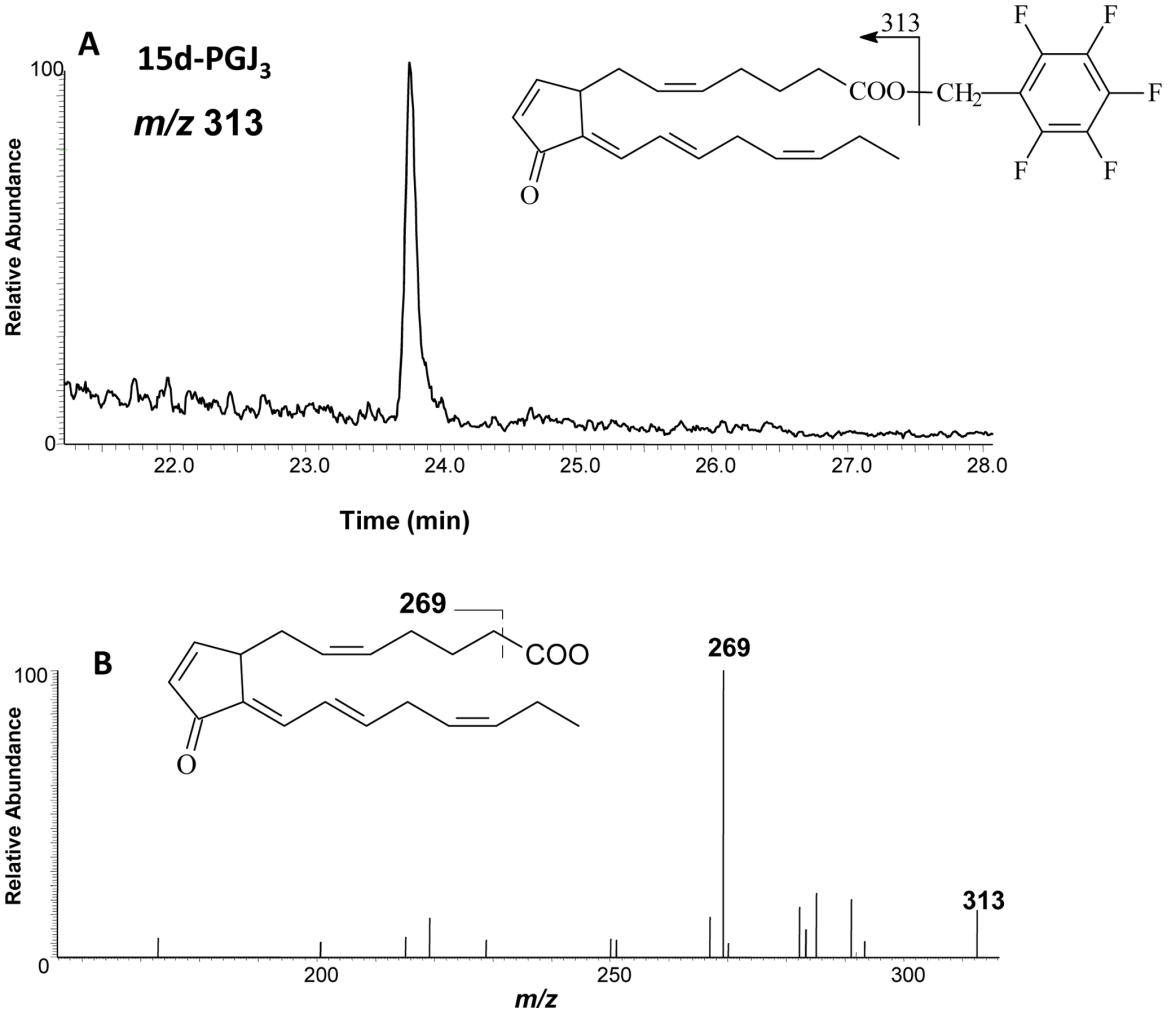
GC-MS/MS analysis of 15d-PGJ_3_ in epididymal adipose tissue of mice fed a EPA-rich diet. 15d-PGJ_3_ was purified by HPLC. The 15d-PGJ_3_ HPLC peak was collected and was analyzed by GC-MS as a pentafluorobenzyl ester derivative (*m/z* 313) (Fig. 8A). B. GC-MS/MS spectrum of 15d-PGJ_3._ The molecular ion [M-CH_2_C_6_F_5_]^-^ of *m/z* 313 was subjected to CID. The structure of the derivative and the proposed structure of the product ion produced by CID of *m/z* 313 are shown in the figure.

We then undertook experiments to determine whether 15d-PGJ_3_ is formed in adipose tissue *in vivo*. Because baseline levels of EPA in animals are very low, we determined if 15d-PGJ_3_ is generated *in vivo* by examining this compound in mice fed with the EPA-enriched diet previously mentioned. A representative GC-MS/MS ion current chromatogram obtained from these analyses is shown in Fig. 8. The chromatogram based on m/z 313 detection represents endogenous 15d-PGJ_3_ (Fig. 8A). The observed production, *m/z* 269, generated by CID of the parent ion [M-CH_2_C_6_F_5_]^-^, is consistent with fragmentation of 15d-PGJ_3_ ([M-CH_2_C_6_F_5_-CO_2_]^-^) (Fig. 8B).

### PPAR-γ Antagonism Decreases PGD_3_- or 15d-PGJ_3_-induced Increase in Adiponectin

**Figure 9:**
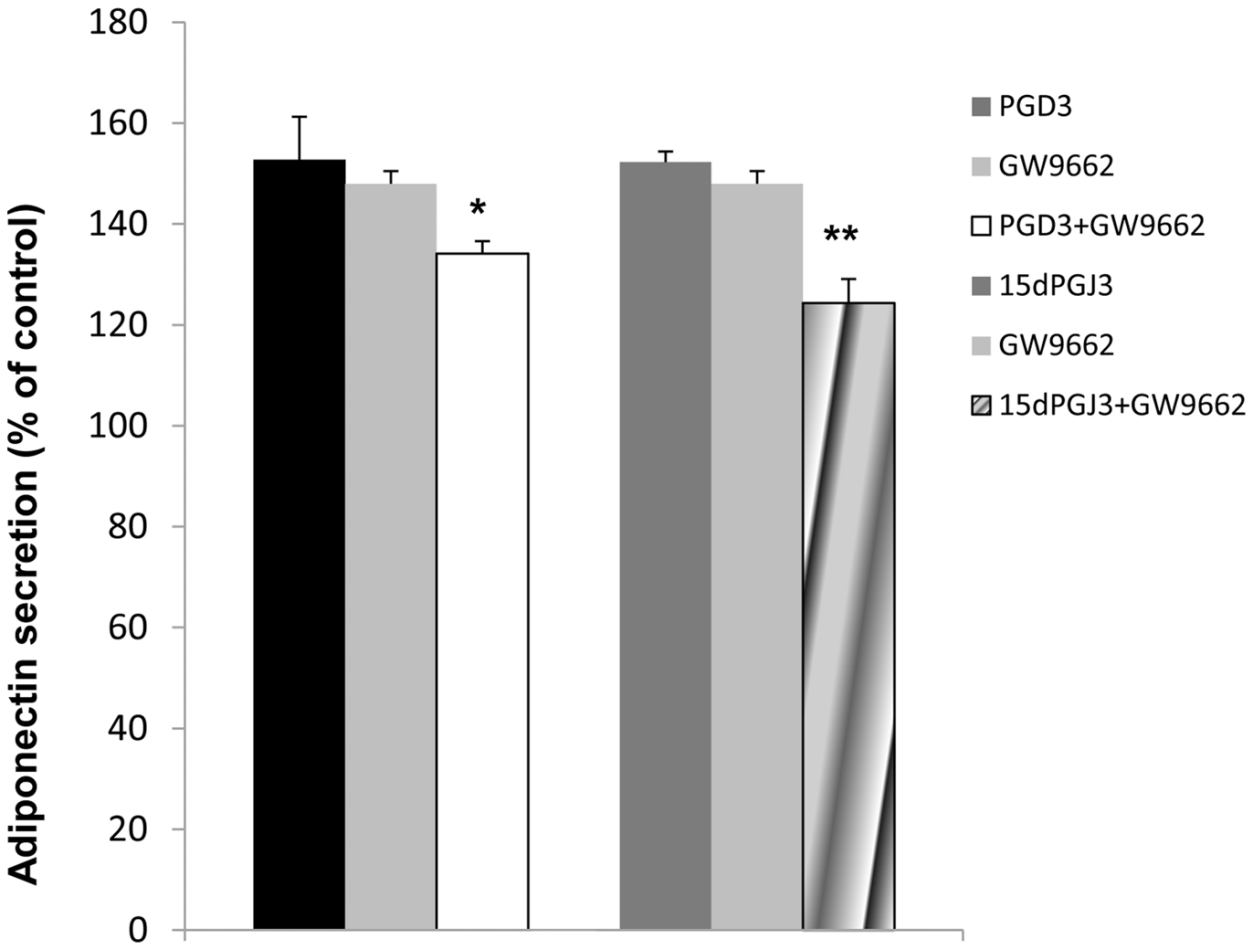
Effect of a PPAR-γ antagonism on the PGD_3_ or 15d-PGJ_3_ increase in adiponectin secretion by cells. 3T3-L1 adipocytes were incubated for 2 h with 1 µM PGD_3_ or 100 nM 15d-PGJ_3_ in the presence or absence of 10 µM GW9662 or with GW9662 alone. Adiponectin in the medium was determined by ELISA. Results are means ± sem (n = 3), expressed as percentage of the control. Statistical significance is represented as *P<0.05, **P<0.01.

Next, we determined whether PGD_3_ or 15d-PGJ_3_ increased adiponectin *via* a PPAR-γ-dependent mechanism. 3T3-L1 adipocytes were incubated with a PPAR-γ antagonist, GW9662, alone or in combination with PGD_3_ or 15d-PGJ_3_. GW9662 alone, which has been previously reported to induce irreversible loss of ligand binding activity [37], had no effect on adiponectin secretion. As shown in Fig. 9, GW9662 significantly attenuated the increase of adiponectin level in cell medium observed in response to PGD_3_ or 15d-PGJ_3_. However, the adiponectin concentration was still significantly increased when GW9662 was added with PGD_3_ or 15d-PGJ_3_ compared with control condition, suggesting that part of the prostaglandin effect was not attributable to PPAR-γ stimulation.

### 15d-PGJ_3_ Mediates Induction of PPAR-γ Target Gene Expression in 3T3-L1 Adipocytes

**Figure 10:**
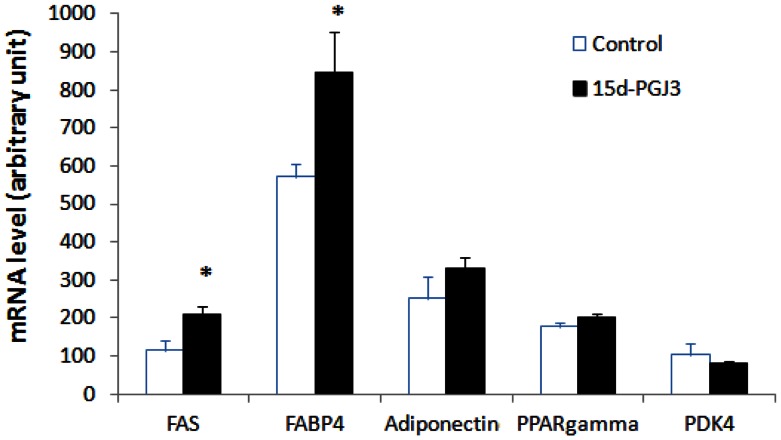
Effect of 15d-PGJ_3_ on FAS, FABP4, adiponectin, PPAR-γ and PDK4 gene expression in 3T3-L1 adipocytes. Cells were incubated for 2 h with or without 100 nM 15d-PGJ_3._ FAS, FABP4, adiponectin, PPAR-γ and PDK4 mRNA levels were quantified by qPCR. Results are means ± sem (n = 3). Statistical significance is represented as *P<0.05 *vs* control.

To go further in the demonstration of the involvement of PPAR-γ, the effects of 15d-PGJ_3_ on the expression of characterized PPAR-γ target genes, FAS, FABP4 and adiponectin were examined. 3T3-L1 cells were incubated with or without 100 nM of 15d-PGJ_3_ for 2 h. As depicted in Fig. 10, 15d-PGJ_3_ induced increase in mRNA level of all PPAR-γ target genes tested with a significant increase in the level of FABP4 and FAS mRNA. However, treatment with 15d-PGJ_3_ had no effect on PDK4 mRNA transcript abundance.

## Discussion

This study demonstrates that EPA increased adiponectin secretion *in vitro* in 3T3-L1 adipocytes. Our finding is consistent with that of Tishinsky et al. [33] who observed stimulatory effects of EPA on adiponectin secretion in human adipocytes but contradicts the finding of Itoh et al. [38] who reported no effect of 200 µM EPA on adiponectin secretion from 3T3-L1 or those of Lorente-Cebrián et al. [39] who showed a significant decreased protein secretion in isolated rat adipocytes treated with 100–200 µM of EPA for 96h. These conflicting data may be explained by the dose of EPA used and the duration of the treatment. Indeed, it has been demonstrated that adipose cells and adiponectin production are sensitive to oxidative stress [40]. Depending on the dose, ω-3 PUFA may have bimodal effect with antioxidant activity at low intake [41] and a prooxidant one at high dose [42]. Furukawa et al. [43] also found that incubation of 3T3-L1 adipocytes with 200 µM EPA for 24h induced an oxidative stress in cells. Thus the exposure to high concentrations of PUFA may lead to lipid peroxidation with subsequent decrease of adiponectin production.

Most studies examining the beneficial effects of ω*-*3 PUFA *in vivo* have used fish oil that usually consist mainly of EPA and DHA [26, 44–46]. Recently, we demonstrated that DHA rapidly increased adiponectin secretion in mice fed a DHA-rich diet compared with mice fed a standard diet [27]. We now show that EPA supplementation also increased adiponectin secretion as early as 4 days after initiation of the EPA-rich diet. The only previous study investigating the effect of EPA on adipocytokine secretion in mice was conducted over a 2-week or 4-week feeding period [38]. The present data show that EPA effect on adiponectin is very fast.

Different cellular and molecular mechanisms have been proposed to explain the beneficial effects of ω-3 PUFA including modification of the membrane structure and the signal transduction [47]–[49], modulation of gene expressions [50], [51]. Another possible mechanism involves the altered pattern of eicosanoid production, eicosanoids derived from ω-3 PUFA having in general anti-inflammatory effects while those produced from ω-6 PUFA being pro-inflammatory mediators [5]. Additionally, in recent years, ω-3 PUFA have been demonstrated to serve as substrates of novel bioactive lipid mediators named resolvins and protectins [6, 7]. We hypothesized that metabolites derived from EPA could also partly be responsible for the increased adiponectin secretion. Our studies have shown for the first time that adiponectin secretion is up-regulated by a novel class of cyclopentenone eicosanoid, 15d-PGJ_3_, issued from PGD_3._

In the present studies, we further explored the molecular mechanism involved in the increased adiponectin secretion by 15d-PGJ_3._ We investigated the role of the nuclear receptor PPAR-γ which is an assumed key transcription factor that regulates the expression of adiponectin [52], [53]. Moreover, the anti-inflammatory effects of the 2-series cyclopentenone prostaglandins have been shown to be mediated, in part, through PPAR-γ [18, 19]. We initially used the GW9662, a covalent PPAR-γ antagonist [54]. We show that GW9662 partially decreased the PGD_3_ and 15d-PGJ_3_-mediated increase in adiponectin secretion. Further analysis indicated that 15d-PGJ_3_ enhanced the expression of transcriptionnal target genes of PPAR-γ, such as FAS and FABP4. Our results suggest that 15d-PGJ_3_ elicits the effect on adiponectin secretion at least partially *via* PPAR-γ. This is consistent with observations showing that 15dPGJ_2_ can act through PPAR-γ-dependent as well as PPAR-γ-independent mechanisms [19, 55–57]. 15d-PGJ_3,_ as 15d-PGJ_2_, is supposed to be particularly reactive, including toward PPAR-γ [58], because of the presence of an highly electrophilic α,?β-unsaturated ketone moiety and two electrophilic carbon centers (at carbons 9 and 13) which readily reacts with substances containing nucleophilic groups. 15d-PGJ_3_ could thus modulate cellular responses through interaction with key intracellular protein targets as it has been shown for 15d-PGJ_2_. For example, it has been reported that 15d-PGJ_2_ directly inhibits the NF-κB-dependent gene expression by covalent binding to a critical cysteine residue in IκB kinase and the DNA-binding domains of NF-κB subunits [56], [57]. The study of Giri and colleagues [59] support the conclusion that the anti-inflammatory actions of 15d-PGJ_2_ include regulation of the PI3K-Akt-NF-κB pathway, independently of PPAR-γ mechanism. In further study, the 15d-PGJ_3_-induced increase in adiponectin through this PPAR-γ-independent mechanism should be elucidated in order to understand the whole mechanism of 15d-PGJ_3_-mediated secretion of adiponectin.

The studies reported herein have characterized the formation of 15d-PGJ_3_ *in vitro* and *in vivo*. We indeed demonstrate for the first time that 3T3-L1 adipocytes can directly synthesize 15d-PGJ_3_ from EPA. The accumulation of significant amount of 15d-PGJ_3_ in the culture medium of 3T3-L1 may be explained by an intracellular production of 15d-PGJ_3_ followed by its excretion to the medium and/or the excretion of PGD_3_ which is then converted nonenzymatically to 15d-PGJ_3_. We also detected 15d-PGJ_3_ in adipose tissue from EPA-fed mice.

In summary, our results indicate that EPA increases secreted adiponectin concentration in 3T3-L1 adipocytes and in mice as early as 4 days after initiation of the EPA-rich diet. We first demonstrate, using 3T3-L1 adipocytes, that prostaglandins of the 3-series formed from EPA also increase the secretion of adiponectin, in part through PPAR-γ-dependent mechanism. This study opens up new avenues for scientific inquiry. This provides the rational basis to explore in depth the production of 15d-PGJ_3_ *in vivo* and its biological activities. This will likely provide important new insights into the role of ω-3 PUFA and their metabolites in physiology and diseases.
